# Multifactorial assessment and targeted intervention in nutritional status among the older adults: a randomized controlled trial: the Octabaix study

**DOI:** 10.1186/s12877-015-0033-0

**Published:** 2015-04-11

**Authors:** Teresa Badia, Francesc Formiga, Assumpta Ferrer, Héctor Sanz, Laura Hurtos, Ramón Pujol

**Affiliations:** Primary Healthcare Centre Martorell, Barcelona, Spain; Geriatric Unit, Internal Medicine Service, Hospital Universitari de Bellvitge, Barcelona, Spain; Bellvitge Biomedical Research Institute, IDIBELL, L’Hospitalet de Llobregat, Barcelona, Spain; Primary Healthcare Centre ‘El Plà’ CAP-I, Sant Feliu de Llobregat, Barcelona, Spain; Support Research Unit, Primary Health Department Costa Ponent, IDIAP Jordi Gol, Barcelona, Spain; Nutrition Unit, Endocrinology Service, Hospital Universitari de Bellvitge, Barcelona, Spain; CAP- Buenos Aires, c/ Mancomunitats Comarcals n°9 .08760 Martorell, Barcelona, Spain

**Keywords:** Elderly, Malnutrition, Intervention

## Abstract

**Background:**

Malnutrition is frequent among older people and is associated with morbi-mortality.

The aim of the study is to assess the effectiveness of a multifactorial and multidisciplinary intervention in the nutritional status among the elderly.

**Methods:**

Randomized, single-blind, parallel-group, clinical trial conducted from January 2009 to December 2010 in seven primary health care centers in Baix Llobregat (Barcelona). Of 696 referred people, born in 1924, 328 subjects were randomized to an intervention group or a control group. The intervention model used an algorithm and was multifaceted for both the patients and their primary care providers. The main outcome was improvement in nutritional status assessed by Mini Nutritional Assessment (MNA). Data analyses were done by intention-to-treat.

**Results:**

Two-year assessment was completed for 127 patients (77.4%) in the intervention group and 98 patients (59.7%) in the control group. In the adjusted linear mixed models for MNA, intervention showed no significant effect during all follow-up period with −0.21 (CI: − 0.96; 0.26). In subjects with nutritional risk (MNA ≤ 23.5 / 30) existed a tendency towards improvement in MNA score 1.13 (95% CI −0.48; 2.74) after 2 years.

**Conclusion:**

A universal multifactorial assessment and target intervention over a two year period in subjects at nutritional risk showed a tendency to improve nutrition but not in the rest of community-dwelling studied subjects. Cognitive impairment was an independent factor strongly associated with a decline in nutritional status.

**Trial registration:**

The clinical trial is registered as part of a US National Institutes of Health Clinical Trial: NCT01141166.

**Electronic supplementary material:**

The online version of this article (doi:10.1186/s12877-015-0033-0) contains supplementary material, which is available to authorized users.

## Background

Over past decades, the oldest group (aged over 80) has been the most rapidly growing population segment, and is expected to reach 10% in developed countries by 2050 [1]. The evidence shows the importance of the role of nutrition in the prevention and postponement of disability in elderly people [2]. Currently, the prevalence of malnutrition in the community of people aged over 65 is 5.8% [3], although a recent study in our cohort showed that 34.5% of community-dwelling 85 year -olds was at nutritional risk according to the Mini-Nutritional Assessment (MNA) [4]. Malnutrition is a well known significant source of morbidity and mortality for older adults [5]. Usually, the reasons for poor nutritional status in older people are multifaceted. So, social, physiological and psychological changes associated with aging are determining factors in nutritional intake in the elderly and have been described as risk factors for malnutrition [6].

In the literature, the effectiveness of nutrition interventions in older people has received growing attention. A recent Cochrane review on food supplements showed that nutritional supplementation produced a small weight gain in older people with no evidence of the potential benefits for an improvement in functional status [7]. Positive results were observed in studies enrolling a selected part of the population who were at high malnutrition risk (for example participants who have experienced recent hospitalization or institutionalized individuals). Most of them have utilized nutritional supplements as the main intervention strategy [8,9], but few have used other approaches [10,11].

Only a few randomized clinical trials [11-13] have used a comprehensive geriatric assessment with a multifaceted risk evaluation of malnutrition followed by targeting interventions at an individual’s risk factors. This is an attractive strategy as it could reduce several components of malnutrition risk and would be expected to lead to greater reductions in malnutrition than would the strategy of dealing with risk factors in isolation [13]. Nevertheless, despite the positive results of these studies, areas remain unexamined [12]. Additional data from long-term multifaceted and individual nutrition interventions trials for older people in the community were still required. Given the heterogeneity of the elderly population in terms of health status, it was hypothesized that individualized interventions and multifaceted risk assessment would be particularly effective in this group. Therefore, the purpose of this study was to consider whether a multifactorial and multidisciplinary intervention model might improve the nutritional status including those with cognitive impairment and co-morbidities in community-dwelling people over 85 years of age. Furthermore, a second objective was to examine whether nutritional strategy could affect clinical, functional and analytical outcomes.

## Methods

### Study design

This study was a randomized single-blind, parallel-group, clinical trial (registration number NCT01141166) of a multifactorial malnutrition intervention in community-dwelling elderly people conducted from January 2009 to December 2010. The design of the study and baseline data has been published in detail elsewhere [4,14]. The institutional Ethics Committee of the Jordi Gol Institute for Primary Care Research approved the study.

### Settings and participants

All community-dwelling inhabitants born in 1924 and registered at one of seven primary healthcare centres in Baix Llobregat (Barcelona, Spain) were contacted. The combined population served by these healthcare teams includes approximately 210,000 inhabitants of a total of 800,000 inhabitants of the Baix Llobregat area (17% being people older than 65). The seven voluntary health care centers involved in other elderly assessments were in the same geographical area and they had similar data regarding immigration percentage (11%) or population served (70%).

The only exclusion criterion was institutionalization (24 hours of professional care available). We did not use exclusion criteria for diseases or cognitive impairment. Research assistants contacted potential participants (by telephone or letter), and those who had no exclusion criteria were asked to participate. All subjects who agreed to participate signed informed consent forms before the study started. People unable to give informed consent were included if they had a relative caregiver who gave informed consent. There were no differences among respondents and non-responders in terms of gender, health care center, or physician in charge.

Data collection was performed of all subjects according to medical records and interviews in primary care services or at home if the subjects were not ambulatory (the caregiver was interviewed, when the elderly person was unable to participate due to compromised health and/or cognitive ability). It was done by a health assistant (medical doctor, or nurse member of health centre), at baseline, and at 12 and 24 months of follow-up.

Gerontology assessment included socio-demographic data such as gender, marital status, education level, presence of caregiver and whether they lived alone. The evaluation of co-morbidity placed special emphasis on diagnosed hypertension, diabetes mellitus, dyslipidemia, ischemic cardiomyopathy, heart failure, stroke diagnosis, atrial fibrillation, dementia, Parkinson’s disease, and previous diagnosis of anaemia. We used the Charlson Index to assess global co-morbidity, which has a minimum score of 0 for a healthy individual and a maximum score of 37 for high co-morbidity [15]. In addition chronic drug prescription was recorded by extensively reviewing prescriptions according to data from medical records and confirmed in personal interviews.

The assessment included sensory status (near vision measured by the Jaeger test and hearing ability measured by the whisper test) [16]. Functional capacity for basic activities of daily living (ADL) was measured by the Barthel Index (BI) [17], which has an ordinal scale from 0 to 100 points (from total dependence in all activities to full independence). We used the Lawton Index (LI) to evaluate the ability to carry out instrumental activities. This index ranges from 0 to 8 points (total dependence to independence) [18]. Nutritional status was evaluated by the MNA, which has a maximum score of 30 points .The MNA consists of 18 brief questions divided into four parts (anthropometric measurements, global assessment, diet information, and subjective assessment). The score (maximum 30 points) allows the following classification: satisfactory nutritional status (>24 points), nutritional risk (23.5 to 17 points) and malnutrition (<17 points) [19]. All participants received a systematic assessment of cognitive impairment and identification of a caregiver. Cognitive status was assessed by the Spanish version of the Mini-Mental State Examination (MEC), which has a maximum score of 35. Scores below 24 reflect cognitive impairment [20]. The social assessment was carried out using the Gijon Social-Familial Evaluation Scale, with a maximum score of 24 points. Scores between 10 and 14 indicate social risk and scores above 15, social problems [21]. Quality of life was assessed using the Life Quality Test (EuroQol-5D) with a Visual Analogical Scale (EQ-VAS) of subjective health, which has test scores ranging from 0 and 100 (where 0 is the worst state of health the patient feels and 100 the best) [22]. The numbers of falls are defined as an unexpected event in which the participants come to rest on the ground, floor, or lower level [23,24]. Hospital admissions and emergency hospital visits in the previous year were also recorded. Health assistants gave participants a self-report: a falls and hospitalizations monthly calendar that was similar to those used in other research trials. A blood sample was collected from each patient after the baseline interview and at 24 months. Blood tests performed included haemoglobin, total cholesterol, HDL cholesterol (HDLc) with low baseline HDLc as <1.0/1.2 mmol/l in men/women, albumin (usual range 35.0-53.0 g/L), ferritin (usual range 21.8-274.7 μg/L), and calcium (usual range 2.2-2.5 mmol/L).

### Randomization

Once the baseline questionnaire had been administered, subjects were randomized to the intervention or control group using a computer-generated randomization table. The allocation was double-blind (to health assistant and evaluator). The researcher who prepared the randomization lists and allocated subjects to study groups had no contact with the study subjects.

**Figure 1:**
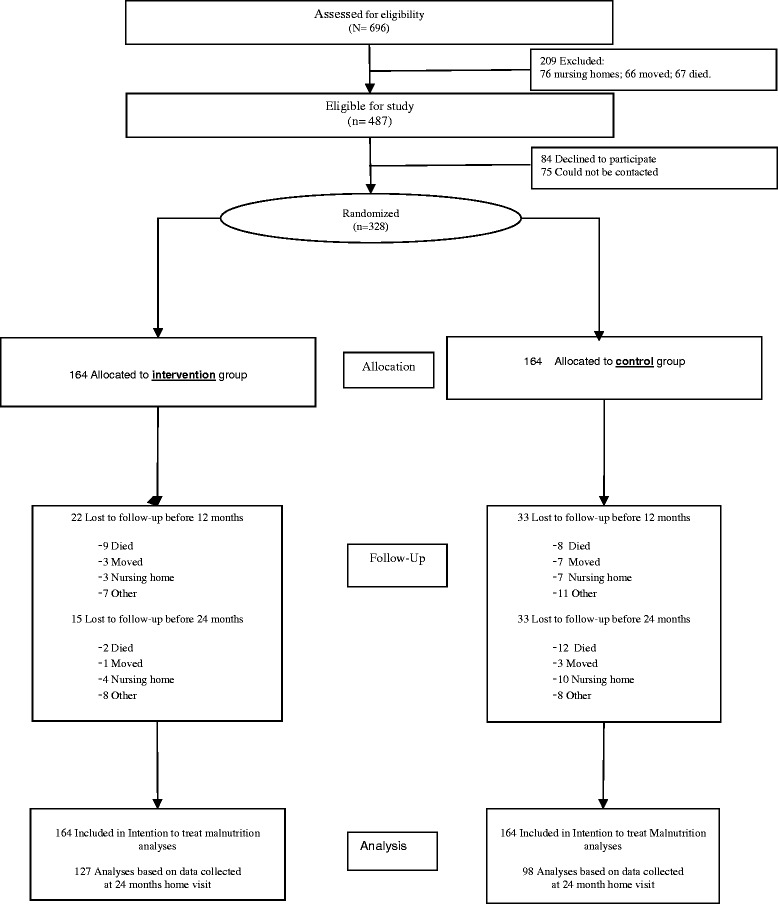
Flow-chart of participants throughout the trial.

Of the 696 subjects screened for the study, 142 did not meet inclusion criteria and 67 died before they were contacted. Of the 487 eligible subjects, 328 (67.4%) were randomized. The loss before randomization was 84 subjects who declined to participate and 75 subjects who could not be contacted (incomplete address, no registered data about death available or moved). Figure 1 Describes the Flow-chart of participants throughout the trial.

### The intervention

Subjects in the intervention group received a community based multifactorial program that links participants to existing medical care and service networks. The intervention was aimed at malnutrition risk factors, and consists of a treatment plan that used a specific algorithm and was multifaceted for both the patients and their primary care provider’s. Additional file 1: Algorithm of targeted risk factors and intervention. The algorithm was applied only to the treatment group identifying nine content areas of potentially modifiable malnutrition risk factors, such as psychotropic and cardiovascular drug use, auditory and vision acuity, balance and gait disorders, nutritional risk, disability, cognitive impairment, social risk and home safety. A health assistant (medical doctor or health center nurse) blinded to treatment assessment, performed one visit each year for two years to intervention participants within 2 weeks after the baseline interview and gave recommendations according to the algorithm.

Participants were advised to contact their primary physician within 1 month to review these results, recommendations, and referrals. The family physician of each participant was mailed after the examination to discuss referrals to medical specialists, medication changes, and follow-up.

The algorithm in the intervention participants evaluated chronic prescription. Special emphasis was placed on the importance of the number of drugs, the progressive discontinuation of benzodiazepines and the use of vitamin supplementation. Subjects were referred to an ophthalmologist when the corrected monocular worst near vision was less than 0.5/1 decimals on the Jaeger chart (<5 Jaeger). If there was any visual field impairment, the patient was also invited to have home modifications to improve visibility using lighting modifications. Participants received nutritional advice on dietary matters and referral to physical therapists for assessment. Information was also reinforced with printed sheets of standard dietary recommendations for the elderly.

The algorithm generated recommendations also for corrected auditory impairment, gait disorders, functional or cognitive decline, home environment barriers and social risk and referral to other medical specialists was initiated when deemed necessary (e.g., referral to a specialist for participants with new cognition impairment or to a cardiologist for participants with new or uncontrolled arrhythmias).

After the initial program session, a health assistant involved in the research intervention assessed its adherence and interim results with a follow-up visit or telephone call every 3 months during the first and second year. The researchers listed any actions that participants reported that they had self-initiated during and after the first session, as well as actions and recommendations that were discussed during the home visit. The additional purpose was to answer questions and to encourage adherence to nutritional advice, physical therapy and exercise, and to other recommendations.

During the second year, two specific interventions were offered: nutritional and rehabilitation assessment. Subjects at nutritional risk (MNA ≤ 23.5/30) received three one-hour individual meetings with a dietician (reference hospital) who developed a plan for individualized nutrition. The content of the meeting included food diary analysis, advice on dietary adaptation to address the most common nutritional problems, recommendations regarding basic cooking techniques, adaptation of textures, or nutritional supplements for patients in need of extra calories. At the end of the sessions, participants received printed information for use at home. On participants with cognitive impairment, the role that played the caregiver was important. They received general information about nutrition, nutritional needs of patients with dementia (recommendations to enrich the diet, improve the texture of food) or other problems related with nutrition in order to assure adherence to the recommendations to the recommendations given. Subjects with one or more falls and no cognition impairment (MEC > 19/35) and ambulation preserved received, over 6 months, four 90-minute sessions with a physiotherapist coordinated by a specialist in Rehabilitation (reference hospital).

To standardize the procedure, the intervention research team received specific training in multidisciplinary geriatric assessment, from a geriatric specialist with experience in educating health professionals for 2-hour sessions a week for 3 months.

The control participants received usual health care which includes a comprehensive general medical consultation when they had a health complaint, limited by a short physician consultation time (7 minutes).

### Follow-up

The primary outcome measure was to assess the effectiveness of nutritional intervention in nutritional status evaluated by the MNA. Secondary outcomes evaluated were the changes over time of diagnosis of anaemia, functional status, analytical parameters and the number of such incidences (hospital admissions and emergency hospital visits). Nutritional status and secondary outcomes were ascertained during the annual assessment and self-reports using the monthly calendar (verified by the health assistant annual assessment) or also by medical record data. The health assistant conducted the 24-month follow-up assessment. In the case of loss of follow-up because of death, the date of death was documented.

Adherence to recommendations was also monitored, using quarterly visits or telephone calls made by the health assistant. A recommendation was adhered to if at least 70% of the session’s program was completed at any time in the 24-month period. To corroborate adherence information, the reports were verified using the primary care medical records (providers computerized agendas, recorded chronic prescription).

### Statistical analysis

For the sample size estimation 30% of prevalence of malnutrition and an expected reduction of 15% was assumed (Relative Risk = 0.5). Assuming 0.10 alpha error and beta error of 0.20 in a unilateral test, the necessary sample size was estimated as 164 individuals both in the control and intervention group. A 50% of replacement was assumed (5% rejecting participation in the study at baseline, 19% of mortality, 6% of drop-out and 20% to compensate for contamination between groups). For the sample size the estimation GRANMO 5.1 was used.

Categorical variables are shown as frequencies and percentages, while continuous variables are presented as means and standard deviations or as medians and interquartile range, depending on normality assumptions (assessed by normal probability plots). In the bivariate analysis for baseline characteristics either the Chi-square or Fisher’s exact test was used to compare categorical variables between the control and intervention group. The Student’s t-test or the Mann–Whitney test, depending on the normality of the variable, was applied to compare continuous variables. As in other studies, we defined two groups: those who were well nourished (MNA > 24) and those at risk of being undernourished, which included people at risk of malnutrition (MNA 23.3–17) and those who were malnourished (MNA < 17) [4].

The primary outcome for malnutrition was analyzed as the evolution of the values of MNA for all follow-up periods adjusting linear mixed models, comparing the control and intervention group. For all models the interaction of the intervention group and time period variable (baseline, 12 months and 24 months) was adjusted in order to take into account differences in MNA evolution between groups. Other models with baseline characteristics of the participants were adjusted. Secondary outcomes were compared between groups as mean differences for continuous variables and incidence for categorical ones on anaemia, functional, analytical and healthcare utilization for the evolution from baseline to 24-month follow-up.

All analyses were carried out by intention to treat. The significance level was set at 0.05. All analysis was performed using R software (Version 2.14.2; The R Foundation for Statistical Computing, Vienna, Austria).

## Results

For the study 328 subjects were randomized. A total of 202 (61.6%) were female, 62 (18.9%) had been educated for more than 6 years and 100 (30.5%) lived alone. Caregivers existed in 174 (53.0%) elderly people. The baseline characteristics of the two groups were similar in terms of age, sex, living alone, co-morbidity and most health-related variables. The geriatric assessment at baseline produced the following mean values: The median Charlson index was 1.00 (0–7) and 199 (61%) subjects was less than 2. The Jaeger test score for median near visual acuity was 5 (3–10), while auditory impairment was present in 124 (37.8%) individuals. In the evaluation of daily activities, the median BI was 95 (85–100) and there were 228 (70%) subjects with IB greater than or equal to 90 and 23 BI individuals scored < 60. The LI median value was 6 (4–8). For cognition, the MEC score was 28 (23; 32), while (238 individuals had MEC values > 23 and 36 (11%) individuals scored MEC < 19. According to the MNA, 215 (65.50%) individuals had correct nutritional status, 101 (30.8%) were at nutritional risk of malnutrition, and 12 (3.7%) individuals had malnutrition at baseline. The median score on Gijón’s scale was 10 (8–11) and 172 (52.4%) subjects was in social risk > 10. The EQ-VAS value for median quality of life was 60 (50–75). The Tinetti scale median score was 8 (5–9). During the previous year, a total of 93 individuals (28.4%) had had at least one fall while 25 subjects (7.6%) had ≥ 2 falls in the past year Table 1. Baseline characteristics of study subjects according to the participation in the intervention group or not. However, subjects in the control group showed high percentage of anaemia (p = 0.03), more ferritin (p = 0.02) and less haemoglobin (p = 0.01). The overall dropout rate was 31.4%. The intervention group had lower rate of withdrawal than the control group: 22.5% versus 40.2% respectively (p = 0.01). Subjects who withdrew from the study showed no significant higher co-morbidity and lower functional, cognitive, nutritional status and quality of life than those who completed the study (data not shown).

**Table 1:** **Baseline characteristics of study subjects according to the participation in the intervention group or not**

**Characteristics**	**Total (n = 328)**	**Control (n = 164)**	**Intervention (n = 164)**	**p-value**
**Gender: female, n (%)**	202 (61.6%)	101 (61.6%)	101 (61.6%)	0.91
**Widowed marital status, n (%)**	174 (53.0%)	85 (51.8%)	89 (54.3%)	0.38
**No education, n (%)**	113 (34.5%)	59 (36.0%)	54 (32.9%)	0.83
**Caregiver, n (%)**	174 (53.0%)	91 (55.5%)	83 (50.6%)	0.44
**Lives alone, n (%)**	100 (30.5%)	50 (30.5%)	50 (30.5%)	0.91
**Hypertension, n (%)**	249 (75.9%)	128 (78.0%)	121 (73.8%)	0.44
**Diabetes mellitus, n (%)**	56 (17.1%)	26 (15.9%)	30 (18.3%)	0.66
**Dyslipidemia, n (%)**	168 (51.2%)	84 (51.2%)	84 (51.2%)	0.91
**Ischemic cardiopathy, n (%)**	20 (6.1%)	6 (3.7%)	14 (8.5%)	0.11
**Heart failure, n (%)**	42 (12.8%)	21 (12.8%)	21 (12.8%)	0.87
**Previous stroke, n (%)**	49 (14.9%)	19 (11.6%)	30 (18.3%)	0.12
**Dementia, n (%)**	31 (9.5%)	17 (10.4%)	14 (8.5%)	0.71
**Anaemia, n (%)**	56 (17.1%)	36 (22.0%)	20 (12.2%)	0.03
**Parkinson’s disease, n (%)**	13 (4.0%)	8 (4.9%)	5 (3.1%)	0.57
**Atrial fibrillation, n (%)**	41 (12.5%)	22 (13.4%)	19 (11.6%)	0.74
Charlson index, median [IQR]*	1.00 [0.0; 2.0]	1.00 [0.0; 2.0]	1.00 [0.0; 2.0]	0.55
Number drugs taken, median [IQR]*	6.00 [4.0; 8.0]	6.00 [4.0; 8.0]	6.00 [4.0; 8.0]	0.50
Visual acuity, median [IQR]*	5.00 [3.0; 10.0]	5.00 [3.0; 10.0]	5.00 [3.0; 10.0]	0.33
**Impaired auditory acuity, n (%)**	124 (37.8%)	58 (35.4%)	66 (40.2%)	0.42
Barthel index, median [IQR]*	95.0 [85.0; 100]	95.0 [80.0; 100]	95.0 [85.0; 100]	0.50
Lawton index, median [IQR]*	6.0[3.7; 8.0]	6.0 [3.7; 8.0]	6.0 [4.0; 8.0]	0.40
**MNA** ^**+**^ , median [IQR]*	25.0 [22.5; 27.5]	25.0 [22.5; 27.5]	25.5 [23.0; 27.5]	0.33
**MEC** ^**±**^ , median [IQR]*	28.0 [23.0; 32.0]	28.0 [22.0; 31.0]	29.0 [23.8; 32.0]	0.16
Gijon test, median [IQR]*	10.0 [8.0; 11.0]	9.5 [8.0; 11.0]	10.0 [8.0; 11.0]	0.68
**EQ-VAS** ^***£***^ , median [IQR]*	60.0 [50.0; 75.0]	60.0 [50.0; 76.2]	60.0 [50.0; 75.0]	0.61
Tineti assessment, median [IQR]*	8.0[5.0; 9.0]	8.0 [5.0; 9.0]	5.0 [2.0; 8.0]	0.60
Number of falls, median [IQR]*	0.0 [0.00; 1.00]	0.0 [0.00; 1.00]	0.0 [0.00; 1.00]	0.53
Haemoglobin, gl mean (SD)**	13.2 (1.57)	13.0 (1.6)	13.4 (1.6)	0.01
Total cholesterol (mmol/L), mean (SD)**	5.0 (1.0)	5.0 (1.0)	5.0 (1.0)	0.88
HDL cholesterol (mmol/L), mean (SD)**	1.4 (0.4)	1.4 (0.4)	1.4 (0.4)	0.66
Albumin, mean (SD)** (usual range 35.0-53.0 g/L)	41.3 (3.9)	41.2 (3.9)	41.5 (3.8)	0.55
Ferritin, mean (SD)** (usual range 21.80-274.0 g/L)	96.1(84.3)	106. 7 (91.3)	86.0 (75.9)	0.02
Calcium, mean (SD)** (usual range 2.2-2.5 mmol/L)	2.3 (0.1)	2.3 (0.1)	2.3 (0.1)	0.32

*[IQR] = interquartile range. **(SD) = standard deviation.. * ***Visual acuity*** *: impaired Jaeger score <5* ***;*** ^**+**^ ***MNA*** *: Mini Nutritional Assessment questionnaire (nutritional risk <23.5) ||* ^**±**^ ***MEC*** *: Spanish version of the Mini-Mental State Examination (cognitive impairment <24);* ***£ EQ-VAS*** *: EuroQol-5D visual analogue scale (0–100).*

**Figure 2:**
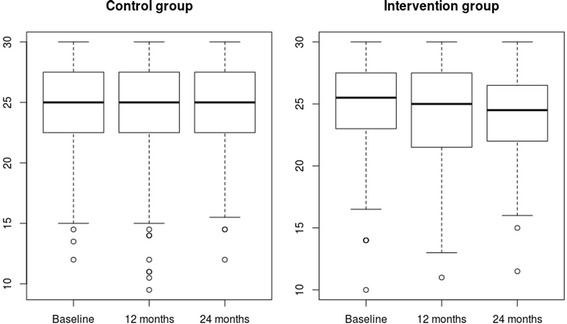
Evolution of MNA values by follow-up periods according intervention group.

The evolution of the MNA by group and follow-up period is shown in Figure 2. Evolution of MNA values by follow-up periods according intervention group. The mean change in MNA score in the 24-month period compared to baseline in the intervention group was −1.0 (−1.7; −0.32) and in the control group −0.62 (−1.4; 0.13) with p value = 0.45 not significantly different between intervention group Table 2. Adjusted linear mixed models taking MNA, intervention treatment showed non-significant effect −0.21 (−0, 96; 0.26) in Model 1. Similar results were found in more complex models as in Model 5 with the coefficient −0.18 and respective 95% confidence interval (−0.85; 0.34). The other adjusted variables in Model 5: women, high co-morbidity, polifarmacy and cognitive impairment were factors influencing the decline in values of MNA with a magnitude of −0.77 (−1.29; −0.18), −0.83 (−1.42; −0.21), −0.70 (−1.34;-0.22) and −2.26 (−2.83; −1.63) respectively.

**Table 2:** **Adjusted linear mixed models taking MNA values for every follow-up period as response**

**Fixed effects**	**Model 1**	**Model 2**	**Model 3**	**Model 4**	**Model 5**
Intercept	24.5	25.8	25.7	25.6	26.3
	(23.4; 25.2)	(24.6; 26.6)	(24.5; 26.4)	(24.4; 26.4)	(25.2; 27.2)
Follow-up period	−0.21	−0.22	−0.22	−0.21	−0.24
	(−0.41; 0.42)	(−0.52; 0.38)	(−0.54; 0.34)	(−0.50; 0.40)	(−0.56; 0.31)
Intervention group	0.61	0.63	0.47	0.64	0.52
	(−0.51; 2.03)	(−0.48; 2.01)	(−0.64; 1.82)	(−0.46; 2.04)	(−0.57; 1.86)
Follow-up period*Intervention group	−0.21	−0.19	−0.19	−0.21	−0.18
	(−0.96; 0.26)	(−0.89; 0.32)	(−0.88; 0.32)	(−0.92; 0.29)	(−0.85; 0.34)
Female	--	−1.18	−0.69	−0.80	−0.77
		(−1.70; −0.56)	(−1.22; −0.13)	(−1.31; −0.17)	(−1.29; −0.18)
Co-morbidity^||^	--	−1.42	--	--	−0.83
		(−2.02; −0.87)			(−1.42; −0.21)
Cognition impairment^§^,	--	--	−2.5	--	−2.26
			(−3.11; 1.89)		(−2.83; −1.63)
Polifarmacy^£^	--	--	--	−1.18	−0.70
				(−1.84; −0.72)	(−1.34; −0.22)
**Random effects**	1.23	1.13	1.02	1.13	0.93
	(0.89; 1.54)	(0.76; 1.47)	(0.58; 1.39)	(0.75; 1.46)	(0.43; 1.33)

Adjusted co-variables are at baseline period. Beta coefficients with 95% confidence interval for every model are shown.

^||^ **Co-morbidity** by Charlson Index (0–37) *≥*2*;* ^§^ ***Cognitive impairment*** *: Spanish version of the Mini-Mental State Examination < 24/35;* **Polifarmacy** ^**£**^ **:** *≥ 4 chronic drugs from medical records*.

Subjects who received specific nutritional intervention (47 participants with MNA ≤ 23.5) showed a non-statistical increase in MNA score with 95% confidence interval of 1.13 (−0.48; 2.74) after one year follow-up (from 12 months to 24 months follow-up). A non-statistical significant decrease in MNA score (−0.32 95% CI:[−1.24; 0.59]) was found in control group participants from first to second year follow-up Table 3.

**Table 3:** **Evolution for secondary outcomes from baseline to 24 months of follow-up Incidence for categorical outcomes is shown**

**Characteristics**	**Control (n = 98)**	**Intervention (n = 127)**	**p-value**
**Anaemia**	13% (6; 22)	11% (6; 18)	0.87
**Barthel index** ^*^	−7.30 (−11.0; −3.7)	−8.40 (−11.3; −5.5)	0.66
Lawton index*	−0.58 ( −0.95; -0.21)	−1.20 (−1.53; -0.88)	0.01
**Hospital emergencies**	30% (20; 42)	23% (15; 32)	0.36
**Hospital admissions**	13% (7; 22)	18% (11; 26)	0.50
Haemoglobin, g/L*	−0.61 (−0.92; −0.30)	−0.26 (−0,44; −0.08)	0.05
Albumin, (normal range 35.0-53.0 g/L)*	−1.30(−1.92; −0.75)	−0.91 (−1.4; −0.42)	0.26
Ferritin, (normal range 21.8-274.7 μg/L)*	−2.83 (−15.5; 9.88)	10.46 (−1.70; 22.6)	0.13

The mean difference between 24 months follow-up and baseline for each intervention group is also shown for quantitative variables. For every estimation the 95% confidence interval is also estimated.

^*^Negative mean differences indicate a decline in the MNA score.

Evolution for secondary outcomes from baseline to 24 months of follow-up Analyses of secondary outcomes yielded a significant difference in longitudinal changes in the control group respect to the intervention group in LI (p = 0.01). The BI, health care utilization and analytical parameters did not show a difference at the 24-month follow-up, although the intervention group showed a significant tendency of a lesser decline in haemoglobin levels compared to patients in the control group (p value = 0.05).

Table 4 Adherence to recommendations in the intervention group at 12 and 24 months of follow-up. The 164 intervention participants received a total of 711 recommendations during the first year and 652 during the second year. Of them 425 recommendations were adhered to in the first year and 522 during the second year. Adherence ranged from 77% (community dietician) to 32% (audiologist assessment), and from 90% (medication and physical therapist) to 40% (community dietician) in the first vs. second year.

**Table 4:** **Adherence to recommendations in the intervention group at 12 and 24 months of follow-up**

**Type of recommendation**	**12 months (n = 150)**		**24 months (n = 136)**	
	**Recommended n (%)**	**Adhered n (%)**	**Recommended n (%)**	**Adhered n (%)**
Discuss medication with primary care physician:	97 (65%)	63 (65%)	102 (75%)	92 (90%)
See ophthalmologist:	88 (59%)	36 (41%)	94 (69%)	75 (80%)
See audiologist:	59 (39%)	19 (32%)	45 (33%)	19 (42%)
See community dietician:	135 (90%)	104 (77%)	109 (80%)	97 (89%)
See community physical therapist:	128 (85%)	95 (74%)	88 (65%)	79 (90%)
See community occupational therapist:	25 (17%)	13 (52%)	39 (29%)	33 (85%)
See neurologist:	34 (23%)	19 (56%)	35 (26%)	29 (83%)
Environmental modifications:	79 (53%)	43 (54%)	85 (63%)	65 (77%)
See social services:	66 (44%)	33(50%)	55 (40%)	33 (60%)
Referred to hospital dietician service:			47 (35%)	19 (40%)
Referred to hospital rehabilitation service:			59 (43%)	30 (51%)

## Discussion

The main result of the present study is that a multifactorial assessment with individual intervention in the oldest old people living in a community-dwelling has not provided evidence of effectiveness in nutritional status. However after receiving individualized nutritional support, subjects included at baseline in the subgroup of “at malnutrition risk” showed improvement in the MNA score. To remark that we found that cognitive impairment is an independent factor strongly associated with a decline in nutritional status.

The Octabaix study included a combined community –dwelling population of oldest old with comorbidities and cognitive impairment. The results of this study showed a group of successful agers with an acceptable health, functionality preserved, good cognition and low comorbidity as published on others studies. Moreover health status of patients living in the community is better than older people living in Nursing homes or Long Term Care Units [25,26].

Nevertheless a Cochrane revision suggest the effectiveness in improving nutritional status in the elderly [7], there is a lack of studies in, oldest old subjects, specialty in the group with poor health status who live in the community [2]. Previous trial nutritional studies in older ages have focused in subjects with a high malnutrition risk with a using single main intervention strategy (dietary treatment or nutritional supplements) with contradictory results [8-11].

In fact, in the present study during the follow up no significant differences in nutritional status were found during the follow up; nonetheless, subjects at malnutrition risk showed a tendency improvement in nutritional status at the end of the second year. These findings are consistent with similar studies in which no significant change in body composition and energy intake was found after individual nutrition counseling and physical exercise in community-dwelling frail elderly people aged 75 and older [27,28]. In contrast the Edit study, an intensive nutritional program, improved nutritional status among malnourished community dwelling patients aged 75 or over older adults [11]. Moreover, a multifaceted approach, with education, training staff support and individual snacks, enhanced and maintained nutritional status over a longer period of time in the elderly living in residential homes although no significant differences were found by MNA. One explanation may be that the MNA instrument measures more factors (global and subjective assessment, diet information) than just weight and is therefore more sensitive to changes [13].

Another reason for the limited effectiveness of this program is the capacity of our national health care system. The education support training of general practitioners is a key to learning about the importance of good care for the health of the elderly [13]. Despite the intervention research team receiving a specific training in multidisciplinary geriatric assessment, the program made referrals to medical specialists through general practitioners who were not trained to address malnutrition intervention. One strategy in the future may be more specific interventions from providers to improve nutritional status, and greater integration of health care services may be required above all to assess, treat and support the elderly.

The third explanation is a lower level of education and subjective health perceptions in the intervention group at baseline. These findings contrast with those described by Kaplan et al. which identified goal setting, motivation, interaction with health care providers and self -reported health perceptions as some of the effective features of nutrition education interventions among older adult participants [29,30]. Therefore, new nutrition interventions, should take into account community-dwelling older adults with less active participation and lower educational attainment to increase motivation and encourage proactive attitudes.

The fourth reason could be that subjects who left the study were often sicker and frailer than those who remained with more co-morbidity and poly-pharmacy. Since the drop-out was higher in the controls than in the intervention arm, then if the sickest and most frail were more often lost from the control group compared to the intervention arm, the remaining controls would have a tendency to be healthier than the intervention group, so the control subjects may be less likely to have developed malnutrition and other adverse events than the intervention group, due to differential rates of loss of the highest at-risk participants.

Another important reason to take into account could be the contamination effect of the control group. Some of the health professionals at the centers involved in the trial had patients in both the intervention and the control group in their practices. The study design did not permit them to know the group allocation of their patients, but when participants came in asking for a medication review or for referral to certain kinds of specialist consultation, practitioners could guess to which arm of the study they belonged. Also there could have been communication among participants. However, this probably would have had a negligible effect [31,32].

Nutritional intervention has been used by other groups and in other conditions; so many studies evaluated and reported a correlation between risk of malnutrition and cognitive impairment [33,34]. Our results showed that the cognitive impairment status was the stronger factor that determines the evolution of the MNA values. Furthermore, the NutriAlz study lowered the risk for malnutrition significantly in elderly people aged 79 and older with dementia living at home with a program managed by a physician and their main caregiver [35]. That suggests the importance of assessing of the difficulties of family caregivers, since some studies showed that the prevalence of poor nutritional status was also found in the family caregiver of elderly people with dementia [36]. Probably nutritional status aggravation is strongly linked to the behavioral disorders of caregivers. The evolution of patients suffering from Alzheimer’s disease is associated with worsening functional capacities with a progressive loss of functional skills in daily living activities [33]. Thus, older people with dementia living at home may experience difficulties in managing their budget, shopping, preparing adequate meals, and recognizing their need to eat. So for the improvement of those disorders to be part of every future longitudinal research applied to the nutritional status it is also important to investigate the role of caregiver.

Functional decline is often multifactorial in older adults and multifactorial intervention strategies might assist in the prevention or postponement of disability. Previous research showed that lower functional status in old nursing home residents may respond better to protein intake [12], and subjects at a high risk of malnutrition improved in functional dimensions mobility and usual activities in a descriptive part of the EuroQol-5D [37]. However, recent reviews do not show positive effects on disability level in the frailest community –dwelling elderly despite an observed effect on total energy intake and weight gain through extra protein and energy [4,38]. Our study found no evidence of improvement in function status. Moreover, the control group showed fewer declines in functional status in carrying out instrumental activities at 24 months than the intervention group. One explanation could be that malnutrition in older years is a slow progressive process- in our study the remaining controls had a tendency to be healthier than the intervention group and instrumental activities changed earlier than the basic activities of daily living. Moreover, home delivered meal programs in old people at risk of malnutrition can impact positively on their ability to perform activities of daily living [39]. One strategy in the future may be to improve the relationship between heath care and community service networks.

The MNA scores have been found to be significantly correlated to nutritional parameters in frail older persons [40]. In our study there was no significant difference between the groups regarding changes in anaemia, serum albumin, and ferritin. Iron deficiency is a common cause of anemia being found in older persons with malnutrition [41]. The same results were shown in an individualized nutritional treatment for 6 months after discharge from acute hospitalization [8], although, unlike what happens in hospitals, in our results the intervention group showed a tendency of lower declines of haemoglobin levels, fewer incidences of diagnosis of anaemia according to medical records and higher deposits of ferritin compared with patients in the control group, in our study a trend of an increase in iron reflected probability improved for diet quality and increase caloric intake compared with the baseline. Probably their baseline and added medical conditions and not only nutritional status influenced the analytical parameters as observed in other studies at community-dwelling older people [42].

Health care use during the study period was compared between the groups. In the literature the decrease in costs of hospitalization related by nutritional status is not proved [9,11], despite reducing the incidence of hospital admissions [9], and neither is the decrease of length of hospital stay [37]. In our study the incidence of hospital admissions was unchanged in the two groups, and there was a trend of a decrease in hospital emergency visits although these differences did not reach statistical significance. The utilization of health care is influenced by different circumstances which are independent of nutritional treatment, so for future studies, larger sample sizes are warranted in order to evaluate the full impact of this model of nutritional intervention.

The strengths of this study are the external generalizability and applicability of the results due to the inclusion of all of oldest-old residents of similar aged registered in the national health care system in one area, regardless of their somatic and cognitive comorbidities.Among the main constraints that should be taken into account, there is a lack of evaluation of the psychological aspects such as depression. Another limitation was that the dietary energy and protein intake were not calculated at the baseline and follow-up, we accepted that MNA score is more representative of the real situation in primary health care, whilst another limitation concerns the high loss of follow-up in this study as in other similar studies, but it resulted in a number of participants that is similar to the median sample of other trials [10].

## Conclusions

In conclusion the present study shows that individually multifaceted multidisciplinary interventions have not consistently shown evidence of their benefit in long-term nutritional status in a cohort of not selected elderly people living at home but, this study highlighted the positive effect on nutritional status in old people at risk of malnutrition. While the impacts of nutritional intervention as well as the best intervention model are still debatable in well-nourished community dwelling participants, there are studies showing that more intense nutritional counseling, specific nutrition intervention, including tailored diet strategies, and nutritional supplement improves dietary intake as well as positive outcomes in the nutritional status or nutrition-related outcomes in elderly at risk of malnutrition or undernutrition living in the community. Future longitudinal studies should emphasize preventive measures in the oldest old people living in a community-dwelling with good nutritional status to prevent undernutrition and specific dietary primary health care programs by medical practice and dietician in patients at risk of malnutrition or malnourished living at the community, and target groups are needed to identify the best treatment model, including new training protocols, new strategies regarding primary care providers, community programs and an investigation of the role of caregiver to improve the assessment of home risk in the most elderly.

## Additional file

Additional file 1:
**Algorithm of targeted risk factors and interventions.**

## Abbreviations

MNA: Mini-nutritional assessment
ADL: Functional capacity for basic activities of daily líving
BI: Barthel index
LI: Lawton index
MEC: Mini-mental state examination
EuroQol-5D: Life quality test
EQ-VAS: Visual analogical scale
HDLc: HDL cholesterol
