# Supplementary material for: Multifactorial assessment and targeted intervention in nutritional status among the older adults: a randomized controlled trial: the Octabaix study
Source: BMC Geriatr. 2015 Apr 11;15:45. doi: 10.1186/s12877-015-0033-0 (PMC4414432; doi:10.1186/s12877-015-0033-0)
Supplement: Additional file 1: — Algorithm of targeted risk factors and interventions. [file 12877_2015_33_MOESM1_ESM.docx]

| **Risk Factor** | **Recommendations** | **Baseline Assessment** | | **3months telephone** | | | **9months telephone** | | | **12 months**  **Assessment** |
| --- | --- | --- | --- | --- | --- | --- | --- | --- | --- | --- |
| **MEDICATIONS:**  .Use of 5 or more medications (over more than 6 months)    . If drugs:        -Anti-inflammatory        -Cardiovascular        -Antibiotics        -Benzodiazepines  -Antidepressants  -Neuroleptics         -Others (diuretics, vitamins) | -Discussion with patient about secondary effects, adherence (Morinsky Test).  -Recommendations to discuss medication with physician to reduce usage of as-needed medication.  - Advice for non-pharmacological options for sleep, pain, constipation |  | | |  | |  | |  | |
| **NUTRITIONAL:**  .If MNA Test <23.5/30  .Problems chewing, swallowing, teething.  .If deficit mouthpiece, bad oral hygiene or not adjusted | -Provide nutrition and exercise booklet  - Exercise 30' before meals.  -Recommendations to physician to consider etiology |  | | |  | |  | |  | |
| **VISION:**  . Visual acuity: Jaeger Tables <5 (20/50)  . D. Mellitus: mydriatic camera review | -Nursing accident prevention advice  -Attention to turning movements ocular-cephalic.  -Have Optometrist or Ophthalmologist. |  |  | | |  | |  | | |
| **SENSE OF HEARING:**  .Whispered voice test abnormal | -Annual review of headphones  -Recommendations to physician to consider etiology and review ORL. |  |  | | |  | |  | | |
| **LOCOMOTOR SYSTEM. BALANCE AND MOBILITY.**  .Join deformity, Callus.  .Tinetti Gait Test (<9)  .Handgryp dinamometry | -Lumbar spine. Upper and lower extremities revision.  -Recommendations for safe performance of mobility tasks.  -Consider referral to physical therapy. |  | | |  | |  | |  | |
| **FUNCTIONAL STATUS**  .Barthel I ≤60/100 | -Patient and caregiver education regarding safe performance task, increased supervision, or other social assistance.  -Recommendations about environmental hazards. |  |  | | |  | |  | | |
| **COGNITION:**  .MEC <24/35 | -Patient and caregiver education avoiding multitasking, review of *diary repercussion, consider depression.*  -Recommendations occupational therapy.  -Recommendations to physsician for further testing or consider neurology advice. |  |  | | |  | |  | | |
| **HOME HAZARDS:**  Carpets, baths, stairs ... | -Removal of throw rugs  -Recommendations for bathroom safety  -Social assistance if necessary to complete assessment |  |  | | |  | |  | | |
| **HOME HELP:**  .Lives alone, social risk revision | - Attention to cleaning, meals, personal care, dependency |  |  | | |  | |  | | |

**Additional file 1:** Algorithm of targeted risk factors and interventions
